# Acupotomy in the management of colostomy stenosis after abdominoperineal resection: a case report

**DOI:** 10.3389/fmed.2026.1748245

**Published:** 2026-07-03

**Authors:** Yun Jiang, Zhenju Liu, Chaoying Cai, Xiaofeng Wang, Yi Yang

**Affiliations:** 1 Department of Proctology, Guang’anmen Hospital, China Academy of Chinese Medical Sciences, Beijing, China; 2 The Second Department of Surgery, Songyang County People’s Hospital, Lishui, China

**Keywords:** abdominoperineal resection, acupotomy therapy, case report, permanent sigmoid colostomy, rectal cancer, stomal stenosis

## Abstract

Stomal stenosis is a well-recognized complication of permanent colostomy following abdominoperineal resection (APR) for rectal cancer, occurring in approximately 2%–15% of patients. Conservative management is the first-line treatment. When conservative treatment fails, surgery is often required, which increases patient discomfort, offers limited therapeutic benefit, and incurs higher costs. We report the use of acupotomy to treat stomal stenosis in a 55-year-old female patient after APR for rectal cancer. Two months postoperatively, the patient developed stomal stenosis. After excluding tumor recurrence, severe infection, and parastomal hernia, acupotomy was performed. Following stoma and peristomal skin disinfection, local infiltration anesthesia with lidocaine was administered. Using the little finger into the stoma as a guide, fibrotic scar tissue at the stenotic ring was carefully released along the bowel wall with an acupotomy knife until the stoma could accommodate an index finger. The stenosis was resolved immediately, and the patient’s symptoms were promptly relieved. Acupotomy may be a feasible, minimally invasive option based on this single case, but the evidence level is limited.

## Introduction

Colorectal cancer is among the three most common malignancies worldwide and represents the second leading cause of cancer-related death (1). Tumors with a distal margin less than 5 cm from the anal verge are classified as low rectal cancers. The abdominoperineal resection (APR), first described by Miles in 1908 (2), has become the standard surgical procedure for low rectal cancer and has significantly improved patient survival. However, the permanent sigmoid colostomy required after APR inevitably impairs the quality of life (3, 4), and stoma-related complications further aggravate patient suffering.

Stomal stenosis is a well-recognized complication of colostomy, with a reported incidence of 2%–15% (5). It can develop at any stage after stoma creation, most commonly due to an overly tight abdominal wall opening during surgery or subsequent peristomal granulation and scar formation. To date, management strategies for stomal stenosis remain limited. Treatment usually begins with repeated dilatation, which is painful and often fails, leading many patients to eventually require reoperation (6).

Here, we present the case of a 55-year-old female patient who developed permanent colostomy stenosis after APR for rectal cancer and was treated with acupotomy therapy. By combining a representative case with a literature review, we aim to enhance understanding of stomal stenosis, explore the potential value of traditional Chinese therapeutic techniques for stomal complications, and propose an integrative approach that may offer clinical value as an option for managing stomal stenosis.

## Case presentation

A 55-year-old female patient was admitted with hematochezia from the abdominal stoma, accompanied by abdominal distension and fever for 1 day. The patient initially presented with hematochezia and was subsequently diagnosed with rectal cancer with synchronous liver and lung metastases in April 2024, confirmed by colonoscopy, pathological examination, and other relevant investigations. She subsequently received three cycles of preoperative XELOX chemotherapy combined with bevacizumab. In June 2024, she underwent laparoscopic-assisted APR with permanent sigmoid colostomy at our hospital. Postoperatively, the abdominal and perineal incisions healed well, the stoma remained viable and pink with good blood supply, and both flatus and stool passage were normal. Three weeks after surgery, she developed intermittent periumbilical and supraumbilical pain. Abdominal radiographs suggested small bowel obstruction, which resolved after conservative treatment, including fasting, gastrointestinal decompression, anti-infective therapy, and nutritional support. The patient denied any family history of hereditary diseases or malignancies. Two months after surgery, she developed abdominal distension and low-grade fever after physical exertion, along with increased flatus in the ostomy bag. At 9:00 p.m. that day, the distension worsened, and by midnight, approximately 10 mL of dark red blood mixed with liquid stool was passed from the stoma. Intermittent passage of similar fluid continued, with a cumulative volume of about 100 mL within 12 h prior to admission. She reported persistent abdominal distension, hyperactive bowel sounds, low-grade fever, and mild fatigue. She denied nausea, vomiting, dizziness, headache, or other symptoms. Her appetite and sleep were poor. On physical examination, the vital signs were as follows: body temperature, 36.6 °C; blood pressure, 99/77 mmHg; heart rate, 99 beats per min; respiratory rate, 18 breaths per min. The patient had experienced low-grade fever (up to 37.8 °C) the day before admission, which resolved spontaneously by the morning of admission The etiology of the transient fever remained unclear. The patient’s height was 166 cm, weight was 55 kg, and body mass index (BMI) was 20.0 kg/m^2^. Abdominal and perineal surgical scars were well healed. The stoma appeared pink and viable, with approximately 10 mL of dark red, blood-tinged fluid mixed with stool in the ostomy bag, along with a small amount of flatus. Abdominal auscultation revealed hyperactive bowel sounds occurring about once every 30 s. Digital examination of the stoma allowed insertion of only the little finger with difficulty, whereas a normal stoma (diameter approximately 1.5–2.5 cm) should easily accommodate an index finger. Circumferential narrowing of the bowel lumen was palpated around the stoma. No significant abnormalities were found on abdominal palpation or percussion. Laboratory results showed white blood cell count of 8.90 × 10^∧^9/L, red blood cell count of 3.58 × 10^∧^12/L, hemoglobin 116 g/L, neutrophil count 6.71 × 10^∧^9/L, neutrophil percentage 75.4%, procalcitonin 0.82 ng/mL, D-dimer 4.66 mg/L, and fibrin degradation products (FDP) 11.7 mg/L. Serum electrolytes, liver function, and renal function were within normal ranges. These findings suggested mild anemia, likely related to the patient’s underlying malignancy and nutritional status. The elevated neutrophil percentage and procalcitonin level indicated a mild inflammatory response, possibly secondary to intestinal stasis and local irritation caused by stomal stenosis. Increased D-dimer and FDP levels were observed; however, these findings were non-specific and may have been related to the patient’s postoperative status, underlying malignancy, or systemic inflammatory response. Contrast-enhanced pelvic computed tomography (CT; Figure 1) revealed marked stenosis of the stomal bowel segment within the abdominal wall tunnel. Although a mild inflammatory response was present, no evidence of severe infection, tumor recurrence, or parastomal hernia was identified. Based on the marked stenosis demonstrated by digital examination and contrast-enhanced CT, the diagnosis of stomal stenosis following sigmoid colostomy was established.

**FIGURE 1:**
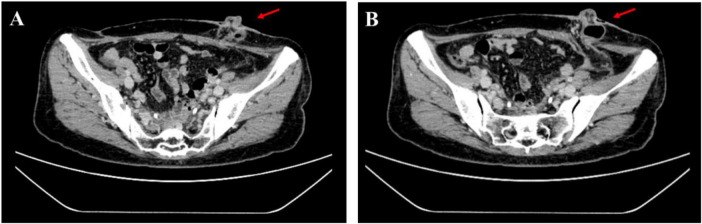
**(A,B)** Contrast-enhanced pelvic CT obtained on admission demonstrated marked stenosis of the stomal bowel segment within the abdominal wall tunnel (red arrows).

The patient was treated with fasting, strict 24-h monitoring of fluid intake and output, antibiotic therapy, and nutritional support. In addition, acupotomy therapy was performed at the bedside. With the patient in the supine position, the stoma and surrounding skin were routinely disinfected. Local anesthesia was administered by injecting lidocaine approximately 3 cm from the stoma edge on both sides. The operator inserted the little finger into the stoma as a guide, and an acupotomy knife (0.6 × 50 mm) was introduced through the left anesthesia site at an angle of approximately 45–60°. Under digital guidance, the position and depth of the acupotomy knife were determined according to the depth of the stenotic ring. Repeated upward lifting and cutting motions were performed from the deepest point until the stenotic ring was palpably relaxed. The same procedure was then carried out on the right side. Throughout the procedure, the acupotomy blade was advanced under continuous digital guidance, maintaining close contact with the operator’s guiding finger. The cutting direction was kept as parallel as possible to the bowel wall tissue rather than forming a significant angle. When resistance at the needle tip indicated that the scar tissue had been reached, cutting was initiated. Each advancement depth did not exceed 1–2 mm. Complete incision of the scar tissue was not required. When the sensation of resistance disappeared or markedly diminished, cutting was promptly stopped and the stoma was gently dilated to minimize the risk of tissue injury. After the procedure, digital examination confirmed that the stoma could accommodate the operator’s index finger (diameter approximately 2.0 cm), indicating complete release of the stenotic ring. The patient reported no significant discomfort during or after the operation. Gentle compression with sterile gauze was applied for hemostasis, and no special dressing was required (Figure 2).

**FIGURE 2:**
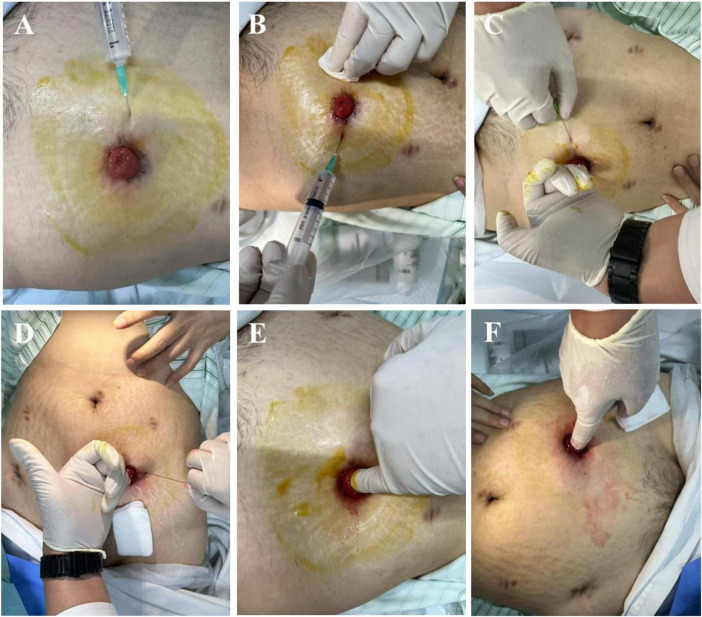
Procedural steps of acupotomy therapy for stomal stenosis. **(A,B)** Local infiltration anesthesia with lidocaine was administered. **(C,D)** The surgeon inserted the little finger into the stoma as a guide, and fibrotic scar tissue along the bowel wall was released with an acupotomy. **(E,F)** Post-procedure digital examination confirmed that the stoma could accommodate an index finger, and the stenotic ring had disappeared.

On the first postoperative day, the patient’s abdominal distension had resolved. Flatus and stool passage were unobstructed, bowel sounds had returned to normal, and digital examination confirmed that the stoma could accommodate an index finger. Her general condition was good, vital signs were stable, and she was able to resume a normal diet. At the 8-month follow-up in April 2025, the patient remained in good general condition. The stoma was patent, and digital examination revealed no evidence of recurrent stenosis (Figure 3).

**FIGURE 3:**
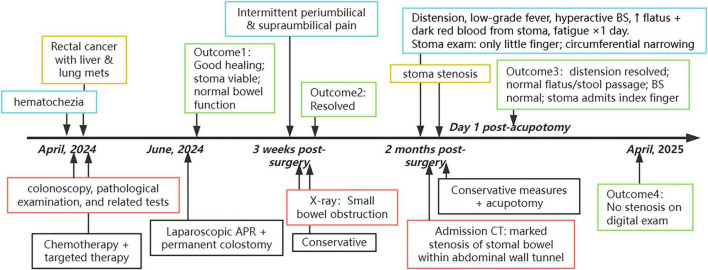
Timeline.

## Patient perspective

I am a 55-year-old woman who underwent surgery for rectal cancer with a permanent colostomy. About 2 months after the operation, I developed stomal stenosis. On my doctor’s advice, I received acupotomy treatment. The procedure was performed at the bedside, was very quick, and I experienced no significant discomfort during or after the treatment. The next day, my abdominal distension had resolved, and both flatus and stool passage were smooth. When the doctor examined my stoma, an index finger could be easily inserted. Moreover, the treatment was very affordable. I am very satisfied with the treatment outcome. Now, 8 months later, my stoma still functions well, and there has been no recurrence of the narrowing. I am very grateful to my doctor for offering me this option.

## Discussion

Stoma formation is often the final step in complex abdominal procedures for both benign and malignant conditions, including inflammatory bowel disease, diverticulitis, abdominal trauma, and colorectal cancer. Although considered a relatively straightforward procedure, stoma formation may be associated with a variety of complications, including stomal retraction, ischemic necrosis, infection, parastomal hernia, stomal prolapse, and stomal stenosis, all of which adversely affect patients’ quality of life (7, 8).

Stomal stenosis is characterized by circumferential contraction at the level of the skin, fascia, or the entire abdominal wall tunnel, leading to progressive narrowing of the stomal lumen. Clinically, it may present as a visibly smaller skin opening with obscured mucosa, or as a seemingly normal stoma in which digital examination reveals a tight, constricted lumen. Typical symptoms include difficulty with stool evacuation, narrowed stool caliber, painful or laborious defecation, abdominal distension, and noisy flatus passage, sometimes accompanied by minor mucosal bleeding. Severe cases may present with signs of partial intestinal obstruction.

Early stenosis is most commonly associated with a small abdominal wall opening at the time of surgery, bowel edema or torsion, or local infection of peristomal tissues. In contrast, late stenosis usually results from mucocutaneous separation, stomal retraction, bowel ischemia or necrosis, or chronic inflammatory changes in the peristomal abdominal wall. These processes eventually lead to secondary healing and scar contracture at the stoma site (9, 10). Stoma creation itself alters the natural route of fecal elimination, causing considerable psychological and social burden, while complications such as stenosis further exacerbate discomfort, embarrassment, and the complexity of stoma care.

Stomal stenosis is among the most disappointing complications of colorectal surgery. Conservative management is the first-line treatment for stomal stenosis. For patients with early mild stenosis after surgery, manual dilation of the stoma is recommended, combined with necessary dietary adjustments and stoma care. When conservative treatment fails, surgical intervention may become necessary, including local revision or stoma reconstruction. Conventional treatments including digital dilation and local revision surgery are effective in appropriately selected patients and are supported by stronger evidence, but they also have varying degrees of shortcomings. Conservative measures are often associated with frequent procedures, significant patient discomfort, short duration of efficacy, and high recurrence rates. Surgical treatment, in turn, is invasive, technically complex, time-consuming, costly, and imposes an additional psychological burden due to reoperation. This case suggests that acupotomy may serve as an additional treatment option for selected patients with stomal stenosis.

Acupotomy therapy is derived from traditional Chinese acupuncture therapy. Based on the “nine classical needles” described in ancient Chinese medicine, acupotomy is a clinical technique primarily used for soft tissue disorders. It was refined and first applied clinically in 1976 by Professor Han-Zhang Zhu in China (11). It combines the therapeutic effects of acupuncture with the characteristics of minimally invasive surgery, using a specially designed instrument with a thick body and a sharp, blade-like tip (Figure 4). Acupotomy is designed to mechanically divide fibrotic, adherent, or contracted soft tissues through a minimally invasive approach. It represents a minimally invasive approach rooted in integrative medicine, offering advantages of simplicity, convenience, and cost-effectiveness (12).

**FIGURE 4:**
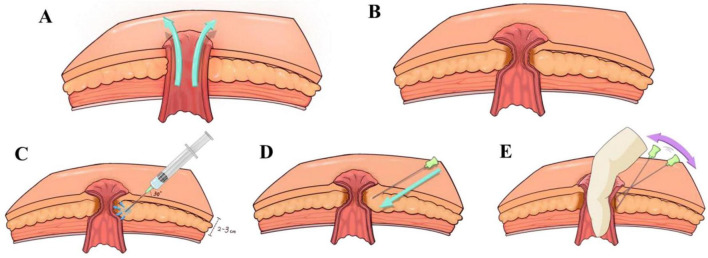
**(A)** Schematic diagram of a normal stoma. **(B)** Schematic diagram of a stenotic stoma. **(C)** Procedure of local infiltration anesthesia with lidocaine injection. **(D)** Procedure of oblique insertion of the acupotomy from the anesthesia site. **(E)** Procedure of inserting the acupotomy into the deepest part of the stenotic ring under digital guidance, followed by repeated superior-inferior cutting until resolution of the ring.

This case presents an early, fibrotic, non-inflammatory stomal stenosis. Acupotomy, a traditional Chinese medical technique, was performed under local anesthesia with stepwise needle insertion to release the stenotic scar tissue surrounding the stoma (Figure 5). Following the procedure, the patient experienced prompt symptom relief, restoration of stomal patency, and no evidence of recurrence during the 8-month follow-up period. Compared with conventional strategies (Table 1), acupotomy may offer several practical advantages in selected patients. It is technically straightforward, performed under local anesthesia, and typically requires only 10–15 min to complete. It is not limited by environmental or logistical constraints. It causes minimal tissue trauma allowing rapid recovery; patients usually resume daily activities immediately without significant pain or anxiety. In this case, symptom relief was observed shortly after the procedure.

**FIGURE 5:**
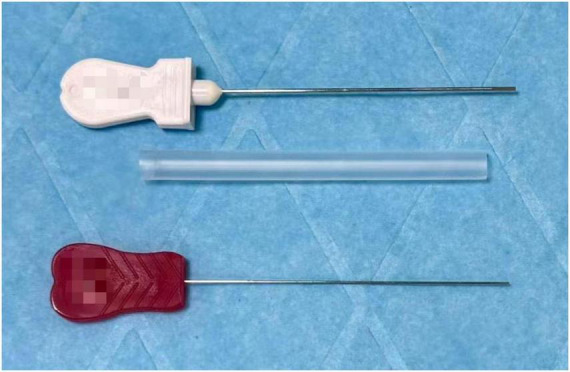
Appearance of the acupotomy knife.

**TABLE 1:** Summary of treatment modalities for stomal stenosis.

Modality		Indications	Advantages	Limitations	Evidence level (14)	Reference
Conservative treatment (first-line)	Digital or Hegar dilator dilation	Early, mild stenosis	Simple; effective in the short term	Requires repeated procedures; mechanical trauma may worsen fibrosis and aggravate stenosis	Level 5 (expert opinion/review)	Bafford et al. (15)
	Rectal tube placement via colostomy for enema and fecal drainage	Obstruction due to stenosis	Provides rapid relief of obstruction; offers sustained efficacy	Recurs after tube removal; increases nursing burden	Level 4 (single case report)	Lv et al. (16)
	Balloon dilation with local triamcinolone injection	Ineffective or recurrent stenosis after digital/simple balloon dilation; patients with severe comorbidities (e.g., cardiac disease, diabetes, multiple prior abdominal surgeries) unfit for stoma reconstruction under general anesthesia	Suppresses inflammation and fibrosis; softens scar tissue; reduces recurrence	Associated with steroid-related risks; lacks large-scale, long-term evidence	Level 4 (single case report)	Matsumoto et al. (17)
	Endoscopic stent placement	Ineffective or recurrent stenosis after digital/simple balloon dilation; patients with severe comorbidities (e.g., cardiac disease, diabetes, multiple prior abdominal surgeries) unfit for stoma reconstruction under general anesthesia	Provides rapid relief; minimally invasive; biodegradable stents eliminate the need for removal	Requires long-term self-dilation; associated with pain, infection, and perforation; lacks large-scale, long-term evidence	Level 4 (case series/case reports)	Issa et al. (18) Kim et al. (19) Aljarad et al. (20)
Surgical treatment	Local revision surgery	Superficial stenosis; failed conservative treatment	Relatively simple; effective in the short term	Prone to recurrence; limited long-term efficacy	Level 5 (expert opinion/review)	Strong et al. (21)
	Stoma reconstruction	Deep or severe stenosis; failed conservative/minimally invasive therapy	Definitive; provides durable outcomes	Invasive; technically complex; prolonged recovery; high complication risk; psychological and economic burden; high risk of recurrence; unsuitable for patients unfit for anesthesia or surgery	Level 5 (expert opinion)	Bafford et al. (15) Aljarad et al. (20)
	Modified W-plasty	Failed conservative therapy or recurrent stenosis; accompanied by difficult defecation or impaired quality of life, without acute intestinal obstruction	Provides good short-term outcomes; improves quality of life; may reduce long-term costs	Technically demanding and time-consuming; risk of parastomal hernia	Level 4 (case report)	Beraldo et al. (22)
	Transposition flap plasty	Moderate-to-severe stenosis	Less invasive; relatively simple; high success rate; low recurrence within 1–2 years	Requires meticulous technique; carries risk of flap necrosis	Level 4 (case series)	Zhang et al. (23)
Traditional Chinese medicine	Acupotomy therapy	Ineffective or recurrent stenosis after digital/simple balloon dilation; patients with severe comorbidities (e.g., cardiac disease, diabetes, multiple prior abdominal surgeries) unfit for stoma reconstruction under general anesthesia	Simple; short procedure; minimal requirements; minimally invasive; enables rapid recovery; low cost	Single case with no control group; short follow-up; lacks large-scale, long-term evidence; safety profile remains uncertain; performed blindly and requires substantial anatomical expertise	Level 4 (single case report, this study)	–

However, this study has several limitations. First, acupotomy was performed as a blind procedure guided only by digital palpation, carrying potential risks of adverse events such as bowel wall perforation, bleeding, hematoma formation, and infection. The safety of this technique depends heavily on operator experience and familiarity with local anatomy, which may limit its generalizability. Therefore, we recommend that the procedure be performed by experienced clinicians after careful preoperative assessment of the stenotic segment. During the procedure, the acupotomy blade should remain under continuous digital guidance and be advanced cautiously along the bowel wall to minimize the risk of tissue injury. If adverse events occur, prompt clinical assessment and appropriate management should be undertaken according to standard surgical principles. Future studies should explore visualized techniques, such as ultrasound-guided acupotomy, to further improve procedural precision and safety (13). Second, the evidence presented in this study is limited to a single case report. This study cannot rule out spontaneous resolution or a placebo effect, and no conclusions regarding efficacy, safety, superiority over standard treatments, or general applicability can be drawn from this report. Moreover, this is only a single case of early, fibrotic, non-inflammatory stomal stenosis, and the range of indications for acupotomy treatment requires further study to avoid overgeneralization. In addition, the follow-up duration in this case was only 8 months, and the long-term recurrence rate remains unknown. Therefore, further prospective studies are needed to evaluate the feasibility, safety, and potential clinical role of acupotomy in the management of stomal stenosis. Third, the assessment of treatment efficacy was primarily based on subjective measures-specifically, the ability to accommodate a finger and symptom relief. Objective quantitative indicators, such as stoma diameter measured by a probe or caliper, or objective evaluation by endoscopic or CT imaging, were not obtained.

## Conclusion

To our knowledge, this is one of the few published reports describing the use of acupotomy for stomal stenosis following abdominoperineal resection. In this patient, acupotomy was technically feasible and was followed by improvement in stomal patency and symptoms. However, given that this report is limited to a single uncontrolled case, no conclusions regarding efficacy, safety, or general applicability can be drawn. Further studies are required to clarify the potential role of acupotomy in the management of stomal stenosis.

## Data Availability

The original contributions presented in this study are included in this article/supplementary material, further inquiries can be directed to the corresponding authors.
